# Antibody-Negative Relapse of Goodpasture Syndrome with Pulmonary Hemorrhage

**DOI:** 10.1155/2019/2975131

**Published:** 2019-04-24

**Authors:** Charlene L. Rohm, Sara Acree, Aseem Shrivastava, Asif A. Saberi, Louis Lovett

**Affiliations:** ^1^Department of Internal Medicine, WellStar Kennestone Regional Medical Center, Marietta, GA, USA; ^2^Department of Pathology, WellStar Kennestone Regional Medical Center, Marietta, GA, USA; ^3^Critical Care Medicine, WellStar Kennestone Regional Medical Center, Marietta, GA, USA; ^4^Department of Graduate Medical Education, WellStar Kennestone Regional Medical Center, Marietta, GA, USA

## Abstract

Goodpasture syndrome is a rare autoimmune disease comprising antiglomerular basement membrane (anti-GBM) crescentic glomerulonephritis and pulmonary capillaritis with circulating anti-GBM antibodies. Rarely, antibody-negative cases have been described. We report a young, African American adult woman admitted with flank pain and hematuria with laboratory testing and kidney biopsy demonstrating anti-GBM crescentic glomerulonephritis with elevated anti-GBM antibody levels. She received treatment but remained dialysis-dependent. She was seronegative and clinically stable until she presented 8 months later with dyspnea and hemoptysis requiring mechanical ventilation. Bronchoscopy revealed diffuse alveolar hemorrhage. She was treated for relapse of Goodpasture syndrome. However, anti-GBM antibodies were undetectable. This case emphasizes prompt diagnosis and treatment of Goodpasture syndrome to preserve renal function. Additionally, clinical manifestations of Goodpasture syndrome and its degree of activity do not necessarily correlate with the actual antibody titer on relapse. Clinicians should have enhanced awareness of this atypical presentation of a rare disease.

## 1. Introduction

The estimated incidence of Goodpasture syndrome is one case per million per year [1]. It is mediated by anti-GBM antibody deposition on the alveolar and glomerular basement membrane confirmed by immunofluorescence. Most patients present with a combination of rapidly progressive glomerulonephritis (RPGN) and pulmonary hemorrhage with a positive anti-GBM antibody titer. However, few cases of antibody-negative relapse of Goodpasture syndrome have been reported. Given the rarity of this disease, both the correct diagnosis of Goodpasture syndrome and the recognition of a relapse are critical in initiating timely and effective treatment. This case describes a patient diagnosed with Goodpasture syndrome who was clinically stable until she re-presented 8 months later with an antibody-negative relapse of Goodpasture syndrome with pulmonary hemorrhage.

## 2. Case Presentation

A 24-year-old African American woman presented to the emergency department (ED) with several months of recurrent nausea, flank pain, and hematuria. She previously sought care 2 weeks prior and was told she had a diagnosis of urinary tract infection (UTI) and was empirically treated with oral ciprofloxacin. However, a urine culture did not grow any organisms, and symptoms persisted despite completing a course of antibiotics. Her past medical history included UTIs for which she received empiric antibiotic treatment, although all urine cultures in her medical chart repeatedly showed no growth of organisms. She was not taking medications. Family history was not significant for any chronic medical illnesses. She smoked cigarettes but denied consuming alcohol or using illicit drugs. On presentation, vital signs were stable and physical examination was significant for bilateral flank pain.

Urinalysis showed red urine, 3 + protein, 3 + blood, 656 red blood cells (RBCs) per high-power field (HPF), 41 white blood cells (WBCs) per HPF, negative nitrites, negative leukocyte esterase, and red cell casts. Blood urea nitrogen (BUN) and creatinine (Cr) were 41 mg/dL and 5.6 mg/dL, respectively, and the glomerular filtration rate (GFR) was 11 mL/min/1.73 m^2^. Urinary protein was 116 mg/24 hours with a fractional excretion of sodium (FENa) of 2.8%. A renal ultrasound demonstrated increased echogenicity of both kidneys suggestive of renal disease. By day 3, creatinine was 6.45 mg/dL and GFR was 10 mL/min/1.73 m^2^. Our patient started hemodialysis.

**Figure 1:**
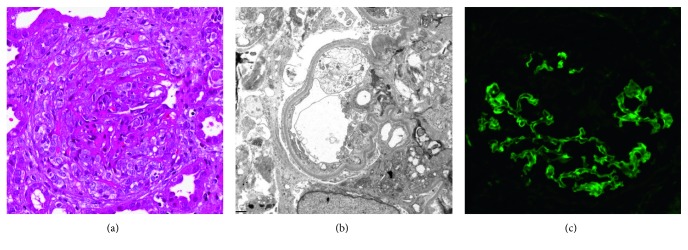
(a) Light microscopy reveals glomeruli with cellular to focally fibrocellular crescent formation and underlying segmental tuft fibrinoid necrosis. Red blood cell casts are noted. There is severe mononuclear inflammation within the interstitium (H&E, 10x). (b) Electron microscopy demonstrates peripheral capillary loops of normal thickness and architecture with widespread effacement of the overlying foot processes. (c) Immunofluorescence microscopy shows strong linear staining along glomerular basement membranes with IgG (4+) and kappa and lambda light chains (3-4+).

Laboratory studies included an antinuclear antibody (ANA), rheumatoid factor (RF), complement C3 and C4, human immunodeficiency virus 1 and 2 (HIV 1 and 2) antibody, antineutrophil cytoplasmic antibodies (ANCA), anti-double-stranded DNA (anti-dsDNA) antibodies, and anti-GBM antibodies. All were negative except for anti-GBM antibodies which reported positive 1 week later with a titer level of 1.7 (<1.0 is normal). In the interim, a renal biopsy was performed due to rapidly declining renal function. Light microscopy showed necrotizing and crescentic glomerulonephritis with fibrocellular crescent formation (Figure 1(a)). Electron microscopy demonstrated widespread podocyte foot process effacement and linear deposits along the glomerular basement membrane (Figure 1(b)) confirmed as IgG by immunofluorescence (Figure 1(c)). A diagnosis of anti-GBM crescentic glomerulonephritis was made.

Before the confirmatory diagnosis was made by renal biopsy, on day 1 of hospital admission, our patient was treated with methylprednisolone 1 g IV daily for 3 days and then prednisone 40 mg by mouth daily for 1 month. Once the renal biopsy confirmed anti-GBM crescentic glomerulonephritis along with serologies positive for anti-GBM antibody, intravenous cyclophosphamide and plasma exchange were both initiated on day 9 of hospitalization. She received cyclophosphamide 1 g IV every 2 weeks for 3 occurrences. She received plasma exchange daily for 7 days then every other day for 3 occurrences. Plasma exchange was stopped once repeat testing for anti-GBM antibodies was undetectable on day 23. Due to rapidly progressing renal failure, she required initiation of hemodialysis on day 4 of admission and continued to require hemodialysis daily throughout her hospital stay. Our patient was discharged to home on day 28 with outpatient nephrology follow-up and hemodialysis three times a week. Anti-GBM antibody levels remained undetectable. Kidney transplant evaluation was initiated.

**Figure 2:**
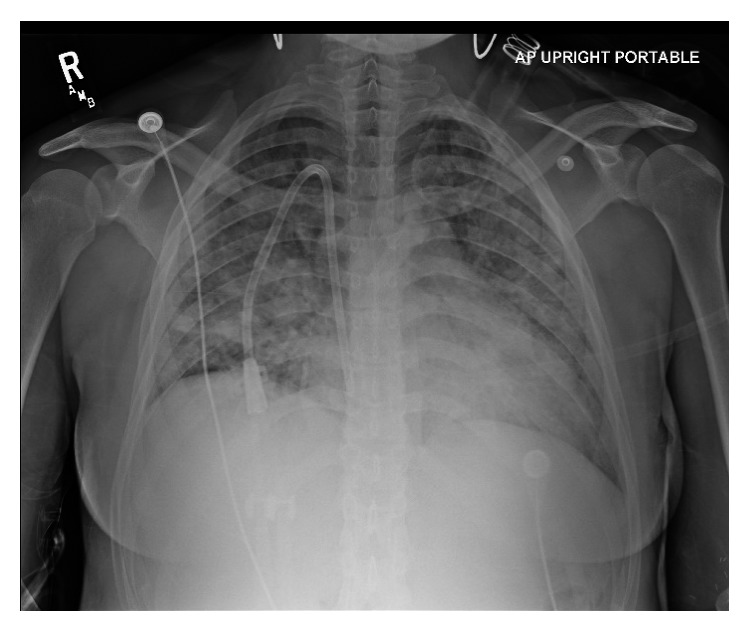
Portable chest radiograph demonstrates diffuse bilateral patchy opacities.

**Figure 3:**
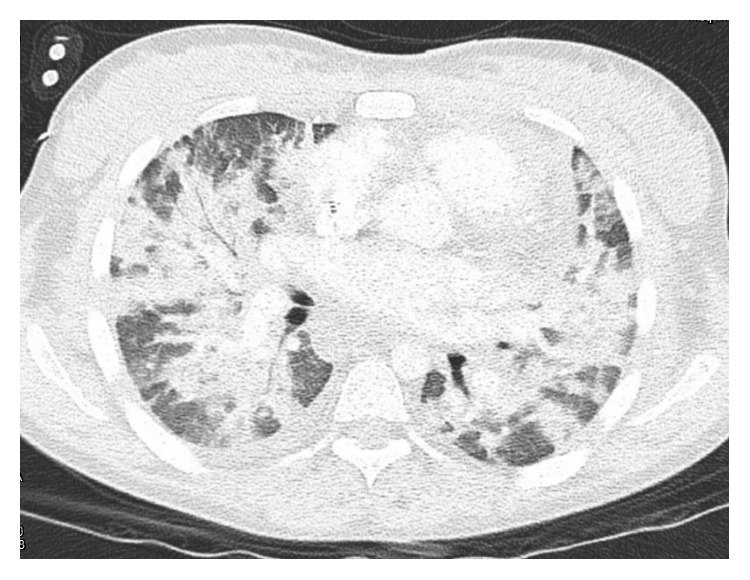
CTA of the chest with IV contrast reveals extensive bilateral consolidations.

Eight months later, our patient presented to the ED with dyspnea and hemoptysis. She last received dialysis the day before admission and had not missed any dialysis sessions. Social history was notable for marijuana use daily for a week prior to admission. She appeared diaphoretic and drowsy with decreasing oxygen saturation despite supplemental oxygen. Physical examination was significant for tachycardia, tachypnea, reduced air movement in all lung fields, and dullness to percussion of the thorax. A portable chest radiograph showed diffuse bilateral patchy opacities (Figure 2). A computerized tomography angiography (CTA) of the chest with IV contrast confirmed diffuse bilateral consolidations (Figure 3). Our patient required tracheal intubation and mechanical ventilation with admission to the intensive care unit (ICU).

**Figure 4:**
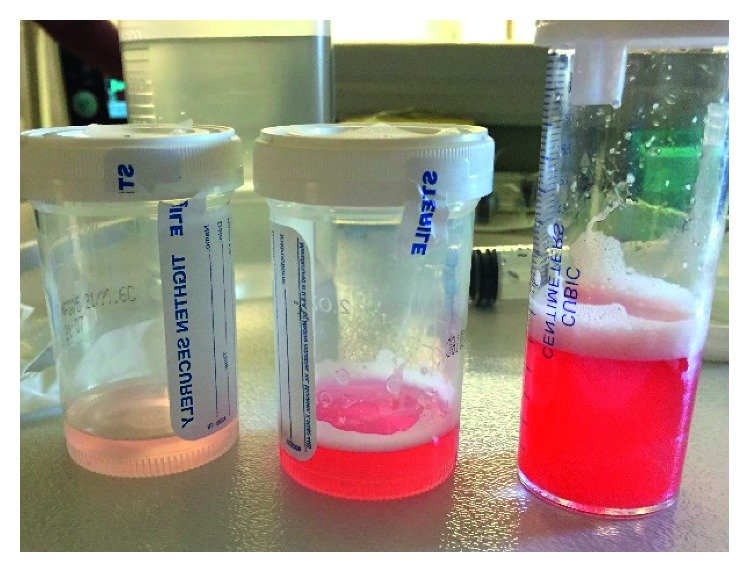
Progressive increase in intensity of blood in sequential aliquots during BAL strongly suggests active alveolar hemorrhage.

Our patient had a hemoglobin level of 6.4 g/dL, hematocrit of 21%, and MCV of 95 fL which dropped to as low as a hemoglobin and hematocrit of 5.6 g/dL and 18%, respectively. Three weeks prior to admission, hemoglobin was 8.7 g/dL and hematocrit was 26%. Flexible fiberoptic bronchoscopy was performed, and the bronchoalveolar lavage (BAL) fluid was consistent with alveolar hemorrhage (Figure 4). The lavage fluid was negative for malignant cells, bacterial, viral, or fungal organisms.

Repeat ANA, ANCA, and anti-dsDNA remained negative. Unlike on initial presentation, repeat anti-GBM antibody levels were undetectable, which did not result until day 7 of the hospital stay. Given our patient's history, a relapse of Goodpasture syndrome with pulmonary hemorrhage was diagnosed. While the most likely diagnosis was a relapse of Goodpasture syndrome, acute heart failure was also included in the differential diagnosis. However, a transthoracic echocardiogram demonstrated a normal ejection fraction of 55–60%, normal left ventricular diastolic function, normal right ventricular systolic and diastolic function, mild tricuspid valve regurgitation with no other valvular abnormalities, an elevated pulmonary artery pressure of 50 mmHg, and a normal central venous pressure. These findings were not suggestive of heart failure; rather, functioning of both left and right ventricles was intact, while the elevated pulmonary artery pressure was suggestive of a primary intrapulmonary process (pulmonary hemorrhage). Starting on day 1, she was treated with methylprednisolone 1 g daily for 5 days, and then changed to prednisone 40 mg daily. Plasma exchange was also initiated on day 1 which she received daily for 5 occurrences. She was treated with mycophenolate 500 mg IV every 12 hours for 5 days. She progressively improved clinically with resolved alveolar hemorrhage and was extubated on day 5. She was transferred to the medical ward in stable condition. On day 10, she was discharged to home with outpatient nephrology follow-up and continued dialysis.

Our patient was stable following her admission for Goodpasture syndrome relapse. She continued to have outpatient hemodialysis 3 times a week. She did not require outpatient oral steroids or immunosuppression. However, 9 months later, we received the news that she had passed away from a drug overdose.

## 3. Discussion

Our patient's urinalysis suggested a glomerulonephritis based on the presence of proteinuria, hematuria, and red cell casts. Subsequently, the diagnosis of RPGN was suspected because of rapidly progressive renal failure. RPGN comprises 3 subtypes: immune-complex glomerulonephritis, pauci-immune glomerulonephritis, and anti-GBM glomerulonephritis (Goodpasture syndrome). Both anti-GBM and immune-complex glomerulonephritis demonstrate glomerular staining for immunoglobulins, while pauci-immune glomerulonephritis has an absence or paucity of immunoglobulins.

Immune-complex glomerulonephritis is associated with systemic lupus erythematosus (SLE), immunoglobulin A (IgA) glomerulonephritis, IgA vasculitis, and postinfectious glomerulonephritis. Our patient had negative ANA and anti-dsDNA antibodies, did not have symptoms suggestive of IgA vasculitis, and was previously healthy with no recent illnesses. Pauci-immune glomerulonephritis is associated with granulomatosis with polyangiitis, eosinophilic granulomatosis with polyangiitis, and microscopic polyangiitis. Our patient had negative MPO-ANCA and PR3-ANCA levels which lower suspicion for their associated diseases.

While the best laboratory predictor of Goodpasture syndrome is serologic detection of anti-GBM antibodies, this serology takes several days to result. Rarely patients can have antibody-negative Goodpasture syndrome so that diagnosis is based on renal biopsy and clinical presentation. Our patient did have a positive anti-GBM antibody level. Light microscopy of her renal specimen showed that 95% of glomeruli had crescent formation. Immunofluorescence demonstrated linear deposits of IgG antibodies along the glomerular basement membrane, which is pathognomonic for anti-GBM glomerulonephritis. Based on our patient's history and physical examination, urinary studies, rapidly progressive disease course, serologies, and renal biopsy, a diagnosis of anti-GBM glomerulonephritis (Goodpasture syndrome) was made.

Eight months later, our patient presented with dyspnea and hemoptysis. The differential diagnosis for hemoptysis and dyspnea is broad, including infectious, immunological, or malignant etiologies. Given the hemoptysis, severe anemia, and chest imaging showing widespread opacities, there was a concern for hemorrhage. Our patient had no clinical history suggestive of an infectious process. Additionally, a BAL fluid analysis did not suggest an infection (no organism seen, rare WBCs, and no bacterial or fungal isolates). A respiratory infection panel which detects a variety of bacteria and viruses in the BAL fluid also returned negative. Negative ANA and anti-dsDNA antibody serologies ruled out rheumatologic disorders. Negative MPO-ANCA and PR3-ANCA serologies effectively ruled out ANCA-associated disorders.

Anti-GBM antibody levels were also undetectable. However, given our patient's history of Goodpasture syndrome, she likely had a relapse of Goodpasture syndrome with pulmonary hemorrhage.

Over 90% of patients with Goodpasture syndrome have circulating anti-GBM antibodies detectable by enzyme-linked immunosorbent assay (ELISA) [2]. ELISA for anti-GBM antibodies is highly sensitive (>95%) and specific (>97%) [2]. All positive results are confirmed by western blotting on collagenase-solubilized human GBM. These assays are rarely negative despite deposition of anti-GBM antibody in the kidney [2]. Although rare, the absence of circulating anti-GBM antibody in the setting of Goodpasture syndrome has been described [3–8]. A review of 6 reports revealed that each Goodpasture syndrome diagnosis had seronegative disease with diagnosis confirmed on renal biopsy. Database review shows that ELISA- and western blot-negative anti-GBM disease may occur in up to 2-3% of patients, requiring renal biopsy confirmation [2]. Therefore, while clinicians may rely on serological detection of circulating anti-GBM antibodies, the early renal biopsy demonstrating linear deposition of immunoglobulin along the GBM with crescentic glomerulonephritis remains the gold standard to diagnose Goodpasture syndrome.

The average time to anti-GBM antibody result reporting for our patient was 7 days since this test was not done in-house. When our patient was first diagnosed with Goodpasture syndrome, she had an elevated anti-GBM antibody level of 1.7. During the subsequent hospital stay for relapse of Goodpasture syndrome, anti-GBM antibody was undetectable by the same commercial ELISA kit BioPlex 2200 Vasculitis (Bio-Rad, Hercules, California). This immunoassay uses as antigen the extracted highly purified human alpha-3 chain of type IV collagen. An indirect fluorescent antibody (IFA) test was not performed. A western blot was not performed.

Prompt diagnosis and treatment of Goodpasture syndrome is critical as it affects the disease course and prognosis. The best predictor of outcomes for crescentic glomerulonephritis is based on serum creatinine and need for immediate hemodialysis. Levy et al. found that if treatment is initiated when serum creatinine is <5.7 mg/dL, patient and renal survival rates at 1 year are 100% and 95%, respectively [9]. Once serum creatinine is 5.7 mg/dL or higher and without need for immediate dialysis, 1-year patient and renal survival decreased to 83% and 82%, respectively [9]. If treatment was initiated when patients presented with dialysis-dependent renal failure, 1-year patient and renal survival was only 65% and 8%, respectively [9]. Our patient may have received an earlier diagnosis of Goodpasture syndrome had her prior urinalyses been more critically assessed for a glomerulonephritis rather than a UTI. Our patient had been told by prior physicians that her recurrent symptoms of nausea, flank pain, and hematuria were due to UTIs, despite multiple urine cultures demonstrating no growth of organisms and no resolution of symptoms with antibiotic treatment. She had incorrect diagnoses of UTI in her past medical history. Her urinalysis showed significant amounts of red blood cells, protein, and red cell casts, which are all suggestive of glomerular disease. Specifically, she had a nephritic syndrome. Furthermore, an FENa of 2.8% and a BUN/Cr ratio of 7 suggested intrarenal damage. When presented with such urinary studies, glomerulonephritis should be suspected. In the setting of a rapid decline in renal function, RPGN is likely. A renal biopsy should not be delayed. Unfortunately in our patient, treatment with plasma exchange and immunosuppression was not initiated until she had developed dialysis-dependent renal failure.

Patients with Goodpasture syndrome typically present with acute renal failure secondary to RPGN. This can be associated with pulmonary hemorrhage. While most patients present with a combination of RPGN and pulmonary hemorrhage, 30–40% present with an isolated renal involvement and 40–60% present with an isolated pulmonary hemorrhage [10, 11]. As in our patient, other cases of relapse with pulmonary hemorrhage have been reported [3, 4, 12–16]. Environmental factors such as cigarette smoking, drug use, and hydrocarbon solvents have been implicated in triggering pulmonary hemorrhage [3, 4, 12–16]. Our patient's ongoing exposure to cigarettes plus her recent use of marijuana may have precipitated a Goodpasture relapse with pulmonary hemorrhage. Relapses resulting in pulmonary disease are strongly correlated with cigarette smoking, which causes inhalation injury leading to alveolar damage. It is possible that subjects with Goodpasture syndrome develop pulmonary hemorrhage when exposed to the right precipitant. Volatile hydrocarbons, cigarette smoking, concurrent infection, high concentration of inspired oxygen, and fluid overload have all been implicated [4].

In conclusion, Goodpasture syndrome is a rare syndrome with variable patterns of presentation. Obtaining a kidney biopsy early and promptly initiating steroids and immunosuppression is critical in preserving renal function and minimizing morbidity and mortality. Goodpasture syndrome must also be considered despite the absence of anti-GBM antibody, as disease activity does not necessarily correlate with antibody titer.

### 3.1. Learning Points

- Critically evaluate urinalysis. Consider glomerulonephritis in the setting of hematuria, proteinuria, and red cell casts.
- Do not delay performing a renal biopsy to definitively diagnose Goodpasture syndrome. Mortality significantly increases once hemodialysis is required.
- Isolated pulmonary hemorrhage can occur as the sole manifestation of relapse in Goodpasture syndrome. This can occur in the absence of detectable antibody levels.
- Current evidence suggests that the clinical manifestations of Goodpasture syndrome and its degree of activity do not necessarily correlate with the actual antibody titer.
